# *Danio Rerio*, the Zebrafish

**DOI:** 10.1002/1097-0061(20000930)17:3<225::AID-YEA34>3.0.CO;2-5

**Published:** 2000

**Authors:** Jo Wixon

**Affiliations:** School of Biological Sciences University of Manchester Oxford Road Manchester M13 9PT UK

## Abstract

The zebrafish has long been a favourite model for the study of vertebrate development. Here we provide an overview of the current state of knowledge and resources for the study of this fish, with comments on the future direction of zebrafish genomics from Professor Mark Fishman and Dr Stephen Wilson.


**Yeast**
*Yeast* 2000; **17**: 225–231.

Featured Organism

### *Danio rerio*, the zebrafish

Jo Wixon
*Managing Editor – School of Biological Sciences, University of Manchester, Oxford Road, Manchester M13 9PT, UK*

### Abstract

**The zebrafish has long been a favourite model for the study of vertebrate development. Here we provide an overview of the current state of knowledge and resources for the study of this fish, with comments on the future direction of zebrafish genomics from Professor Mark Fishman and Dr Stephen Wilson. Copyright © 2000 John Wiley & Sons, Ltd.**

### Summary

- **3 cm long, translucent, teleost fish with black stripes running along their length.**
- **Eggs are fertilized and embryos develop (in 2–4 days) externally.**
- **Embryos are clear and easily observed under the microscope.**
- **Short generation time (~3 months) is ideal for genetics.**
- **Diploid, ~1700 Mb genome, as 25 chromosomes.**
- **25 linkage groups with centromeres have been characterized.**
- **Radiation Hybrid and genetic maps, YAC and P1 libraries and EST resources available, large-scale mutagenesis project completed, second round under way.**
- **Comparative studies with other fish, in particular medaka and pufferfish, and other vertebrates, mainly with human and mouse.**

### Background

Zebrafish (*Danio rerio*) are small, ~3 cm long. They are native to streams in India and are commonly kept as pets. The males are slender and torpedo-shaped, with black longitudinal stripes and usually a gold colouration on the belly and fins. Females are fat when laden with eggs and have little, if any, gold on their undersides.

Zebrafish are easy to raise, with a short generation time of 3 months, and the females can lay hundreds of eggs at weekly intervals. Fertilization is external, allowing easy access to embryos for observation and manipulation. Developing embryos are easily studied under a dissecting microscope since they are transparent. Zebrafish embryos develop rapidly (in 2–4 days), with a beating heart and visible erythrocytes by 24 h. Another advantage of studying the zebrafish is that, in contrast to other fish, which can be triploid or tetraploid (which makes genetic analysis difficult), it maintains the diploid state.

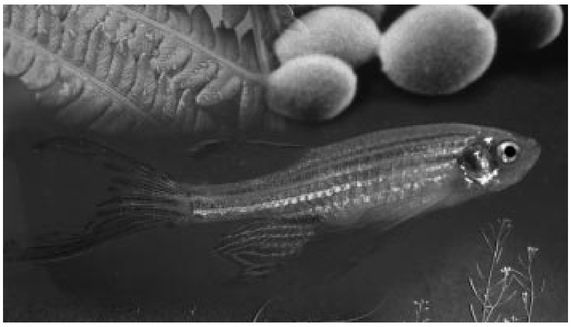

The zebrafish has been shown to be a useful model for the development of several complex tissues such as the kidney (Drummond, 2000), the olfactory system (Ardouin *et al.*, 2000) and the visual system (Saszik *et al.*, 2000). In the study of haematopoiesis, zebrafish mutants have been observed to demonstrate strikingly similar phenotypes to those in human diseases (Amatruda and Zon, 1999).

### Sources

The K12 page: **http://www.neuro.uoregon.edu/k12/zfk12.html**
The Zon lab page: **http://genetics.med.harvard.edu/~zonlab/**

Copyright © 2000 John Wiley & Sons, Ltd.

The Stainer lab page: **http://www.ucsf.edu./dyrslab/zfish.html**

### Tools for study

Despite the lack of a technology for gene knockout by homologous recombination, zebrafish researchers have amassed huge numbers of mutant lines. The bulk of these were created in a large-scale mutagenesis project using the chemical mutagen, ethylnitrosourea (ENU), followed by screening for developmental defects (Haffter *et al.*, 1996; Driever *et al.*, 1996). ENU has the advantage of generating small mutations (compared with those caused by irradiation), which often affect only one gene. These studies revealed mutants defective in the development of many organ systems, such as the retina (Malicki *et al.*, 1996), the inner ear (Whitfield *et al.*, 1996) and the cardiovascular system (Stainier *et al.*, 1996). After exhaustive study of the mutants produced from these experiments, it became clear that saturation had not been reached and the initiation of a second large-scale mutagenesis screen for zebrafish, the 'Tübingen 2000' screen, was announced recently. The screen will be undertaken by Artemis Pharmaceuticals in collaboration with labs at the Max-Planck-Institutes of Developmental Biology and Immune Biology, the University of Heidelberg, the Howard Hughes Medical Institute in Boston and University College London (see this issue, pp. 232–234, for an interview with Dr Stefan Schulte-Merker of Artemis Pharmaceuticals on the mutagenesis screen).

A zebrafish radiation hybrid (RH) panel (commonly known as the 'Goodfellow' panel; Kwok *et al.*, 1998) was produced. This has been used as the basis for a map designed to aid the positional cloning of the genes mutated in the strains from the mutagenesis screens (Geisler *et al.*, 1999).

The NIH Institute-wide Zebrafish Genome Initiative, overseen by the Trans-NIH Zebrafish Coordinating Committee, was started in September 1998. A YAC library has been created, which consists of 17 000 clones, and provides ~five-fold coverage of the zebrafish genome (Zhong *et al.*, 1998). There is also a P1 library, which consists of 4–5 genome equivalents, with clones having an average insert size of 115 kb (Amemiya and Zon, 1999). The initiative is also funding the creation of a 0.5–1.0 cM microsatellite map (Shimoda *et al.*, 1999), mapping of the 'Goodfellow' and 'Ekker' (Hukriede *et al.*, 1999) radiation hybrid panels, which are to be populated with ~10 000 expressed sequence tags (ESTs), and a meiotic map (Kelly *et al.*, 2000), which is to include markers in common with the RH maps. To supply those creating the maps with markers, there is also funding for the generation of ~100 000 ESTs from oligo-fingerprinted cDNA libraries representing several zebrafish embryo developmental stages and different tissues. Another project in the Genome Initiative is to create deletion mutants of the entire genome. This uses an approach in which $\gamma$-irradiated sperm are used to fertilize wild-type eggs (Fritz *et al.*, 1996). DNA from gynogenetic haploid progeny of females produced from this fertilization is used as the template in multiplex PCR with primers for any chosen locus. Females carrying the required deletion are identified due to failure to amplify that locus in a fraction of their progeny. The mutants produced will be placed in the Zebrafish Stock Center at the University of Oregon, Eugene (see Web-based Resources), which is also supported by the NIH initiative.

Tools available for functional analysis in zebrafish include a selection of ectopic expression vectors (Hyatt and Ekker, 1999), with the option of having the gene of interest under the control of tissue specific or inducible promoters (Lekven *et al.*, 2000). One particular example, which allows targeted gene expression in the zebrafish, is called Gal4-UAS (Scheer and Campos-Ortega. 1999). This system involves two vectors, which are used to create separate transgenic zebrafish lines. The first carries the yeast Gal4 transcriptional activator, under the control of a given promoter; the second carries the gene of interest fused to the Gal4 DNA-binding motif. The zebrafish are then crossed to achieve spatial transcription of the chosen gene. Being transparent, zebrafish are ideal for expression pattern monitoring using *lacZ* or *GFP* fusion constructs. Alternative overexpression systems can use RNA or DNA injection (Krauss *et al.*, 1993; Linney *et al.*, 1999) and even large clones such as BACs have been used to rescue mutant phenotypes (Yan *et al.*, 1998). This type of approach is crucial in identifying the gene in a chosen mutant that is responsible for the observed phenotype.

A recent addition to the battery of techniques for knocking out gene function in zebrafish is RNAi. This strategy has been used in *Caenorhabditis*

Copyright © 2000 John Wiley & Sons, Ltd.

*elegans*, *Drosophila* and mouse and has now been shown to work in zebrafish (Li *et al.*, 2000; Wargelius *et al.*, 2000). If this can be shown to work reliably, it could open an exciting new avenue for zebrafish genomics, in which a null phenotype can be achieved for a chosen gene of interest. Another similar approach is to block gene expression using morpholino antisense oligonucleotides. These modified oligonucleotides form more stable triple-helices with DNA than normal oligonucleotides (Lacroix *et al.*, 2000). As yet, though, no in-depth study of this method in zebrafish has been published.

### Current Status of Genome Knowledge

The zebrafish genome is $\sim$1700 Mb in size, as 25 chromosomes. The basis for all of the maps of the genome consists of 25 linkage groups on which the 25 centromeres have been placed (Johnson *et al.*, 1996). There are radiation hybrid (RH) and meiotic maps of the genome onto which CA repeat markers, mutations and ESTs have been placed. There are currently 3000 microsatellites and over 1000 genes and ESTs on the meiotic map (Kelly *et al.*, 2000), and 3000 ESTs have been placed on the 'Goodfellow' RH panel, 1000 of which have also been mapped on the 'Ekker' RH panel (Zon Lab web page).

Comparison of the zebrafish gene map to a selection of mammalian genomes (Postlethwait *et al.*, 1998) yielded significant evidence of synteny. A comparison of the zebrafish *HOX* clusters to those of other vertebrates (Amores *et al.*, 1998) provided evidence for a genome duplication event, which is thought to have occurred after the divergence of ray-finned and lobe-finned fishes but before the teleost radiation. Further comparison of the seven zebrafish *HOX* complexes with the four in *Fugu* confirmed gene loss as a dominant feature of the evolution of tetrapod and teleost *HOX* clusters (Aparicio, 2000).

### Future aims

Professor Mark Fishman is the director of the Cardiovascular Research Center at the Massachusetts General Hospital. His group is applying a genetic approach to the study of the earliest steps of development of cardiovascular form and function in zebrafish.

He feels that the key to the zebrafish is the genetic screen. He did the first large-scale screens in collaboration with Wolfgang Driever (and in parallel with Professor Christiane Nüsslein-Volhard's group) and they were remarkably informative. They were astounded by how modular the genetic disruptions were, i.e. that single gene mutations deleted or perturbed single units of form (e.g. removing a chamber or a valve of the heart). This demonstrated the unitary logic of organ assembly. The next step will be to clone these genes, which will provide the necessary handles on molecular pathways for these units, such as the 'ventricular pathway'.

A second consideration in his work is that organs are functioning devices, and embryonic survival depends upon certain of them, such as the cardiovascular system, kicking in from day one. The zebrafish is quite viable and vigorous without a functioning cardiovascular system because it survives by diffusion from the water during the first few days. This is in distinction to the mouse, in which secondary degeneration begins immediately upon cessation of cardiac function. Thus, for the first time, it is possible to analyse the genes that drive function in the embryo. So, he can now see a way to answer questions like: 'What makes the first heart beat?'; 'How does contraction drive cellular development?'; and 'Does blood flow cause or modify vessel formation?'. He also sees similar questions being answered with regard to the onset of function for renal, gut and other organ systems. The phenotypes of many of these mutations provide eerily accurate phenotypic models of complex human diseases, for which we have few candidate genes. In this regard, he feels that the zebrafish will be the salvation of physiology and the way to search the human genome project for real gold.

Professor Fishman's view is that the main value of mapping and sequencing of the genome is that it will expedite mutation cloning. However, he notes that zebrafish genome comparisons with other species will provide good starting points for analysis of regulatory elements, and insight into how their divergence might cause large-scale structural changes during evolution.

He anticipates that new screens will be applied to the understanding of organ formation and physiology, evolutionary biology and other areas. Behaviour, learning, and memory are already the focus of some early phase studies and suppressor and

Copyright © 2000 John Wiley & Sons, Ltd.

enhancer screens will help refine pathway descriptions. In addition, the relatively ready penetrance of many chemicals into the viable embryo makes them targets for chemical screens, relevant to drug discovery and toxicology.

Dr Stephen Wilson is a Wellcome Senior Research Fellow and Reader at the Department of Anatomy and Developmental Biology at University College London. His group is studying the mechanisms underlying the patterning of cells and tissues in the embryonic zebrafish, with a focus on the embryonic development of the forebrain.

He feels that genome sequencing is a major priority for the community for several reasons, but perhaps primarily to facilitate the rapid cloning of genes affected by mutations isolated in a wide variety of genetic screens, using a candidate gene approach.

He sees full-length cDNA sequencing as an essential parallel project to genome sequencing to facilitate the identification of coding sequences within the genome. This will be essential to aid in the cloning of mutations and for functional analysis of the genome, as a wide variety of mis-expression strategies now exist in zebrafish, provided that one has access to full-length coding sequence. Generation of new libraries, from wild-type and transgenic lines, is likely to be essential for this project. As further cDNA resources become available, such as Unigene sets, then the generation of extremely well-characterized arrayed libraries will be possible.

He also feels that zebrafish are perhaps the most suitable vertebrate model system (owing to their transparency and availability in huge numbers) in which to perform large-scale expression profiling analysis of genes by *in situ* hybridization. Using whole embryos at a variety of developmental stages, it is possible to rapidly, accurately and economically determine sites and times of expression of thousands of genes during embryonic and larval development. This type of analysis has been initiated in several groups, but Stephen's feeling is that it needs to be expanded and funded on a larger scale.

From these large-scale expression profiling projects, large numbers of genes with highly specific expression patterns will be isolated. In his opinion it will be important to establish selected transgenic GFP lines that label specific cell populations in the fish by using regulatory sequences that control the expression of these genes. Such lines will be essential for detailed phenotypic analysis (by crossing them to mutant lines), for detailed screening procedures and for the isolation of cell type-specific RNA populations that may be underrepresented in more general libraries.

Stephen points out that if antisense morpholinos turn out to reliably inhibit gene function, then as soon as full-length cDNA sequences are obtained, large-scale 'knock-out' analysis becomes possible. This would certainly not replace true genetic approaches, but may be an initial first indicator of gene function. He also notes that existing screens have isolated only a fraction of the mutant lines that can be obtained and so it is clear that further directed screens are essential. These should include screens by morphology, by gene and protein expression (using wild-type and transgenic-GFP lines), by behaviour, physiology and as many other criteria as investigators can establish.

In addition, Stephen asserts that more knowledge of anatomy, physiology, cell biology and behaviour of developing and adult fish, organized and presented on the web via ZFIN (see Web-based Resources), will be required to fully make sense of gene function analysis studies.

### Web-based resources

### General information on zebrafish

**The Zebrafish Information Network (ZFIN)**
**http://zfish.uoregon.edu/**
This page contains a vast array of information on zebrafish anatomy, genetic strains, maps, genes, ESTs and probes. There are news pages, a protocol book, a link to the zebrafish newsgroup, laboratory and researcher lists and information about the stock centre.

### Mapping and sequencing projects

**The Zebrafish webserver at the Cardiovascular Research Centre of Massachusetts General Hospital**
**http://zebrafish.mgh.harvard.edu/**
This site provides access to zebrafish genetic maps, made using microsatellites, as a searchable on-line database and figures of the maps of each of the 25 linkage groups. There is also an atlas of zebrafish anatomy and information on a YAC library created by the group.

Copyright © 2000 John Wiley & Sons, Ltd. 

**The WashU Zebrafish Genome Resources Project**
**http://zfish.wustl.edu/**
The group are developing ESTs from ~50 000 zebrafish cDNAs, derived from libraries produced from a selection of sources, such as adult brain, kidney and liver. The cDNA clones are available from Incyte (**http://www.genomesystems.com**) and from the Resource Centre/Primary Database (RZPD) section of the German Human Genome Project (**http://www.rzpd.de**). The project also involves mapping of the ESTs onto a radiation hybrid (RH) map.

**The Children's Hospital Zebrafish Genome Initiative**
**http://134.174.23.167/zonrhmapper/Default.htm#index**
This page provides maps and a service in which the group will use your sequence or your primers to place a marker of your choice onto the map. They also provide the protocols they are using so that users can map a marker onto the panel themselves.

**The Dawid Laboratory – Zebrafish Radiation Hybrid Mapping**
**http://dir.nichd.nih.gov/lmg/lmgdevb.htm**
This group provides access to their radiation hybrid panel so that users can place a marker of interest onto the map. There are images of the maps of all 25 linkage groups and mapping data from a cDNA expression screen.

**The Trans-NIH Zebrafish Initiative**
**http://www.nih.gov/science/models/zebrafish/**
This page contains information on funding opportunities, reports of previous meetings relating to the initiative, future meetings and courses and a list of zebrafish links.

### Gene indices

**The TIGR Gene Index for Zebrafish**
**http://www.tigr.org/tdb/zgi/**
This is a database of zebrafish expressed sequences classified into three categories: ESTs (partial, single-pass sequences from either end of cDNA clones); CT's (tentative consensuses, built from overlapping EST sequences); and ETs (mature transcripts, with untranslated regions where possible). The database can be searched with a gene name or identifier, by tissue or library identifier, or by using a BLAST search with a sequence of interest.

**The NCBI Unigene Zebrafish Sequences Collection**
**http://www.ncbi.nlm.nih.gov/UniGene/Dr.Home.html**
A non-redundant set of zebrafish genes generated from known genes and automated clustering of the EST data. These can be searched by keyword or cDNA library type.

### Zebrafish stock centres

**The Zebrafish International Resource Center**
**http://zfish.uoregon.edu/zf_info/stckctr/stckctr.html**
The center has a collection of wild-type, mutant and transgene-carrying fish and frozen sperm. The center provides information and advice on keeping zebrafish and researchers can visit the center to learn techniques or screen the collection for mutants of interest.

### Comparative and functional genomics projects

**The Stanford Zebrafish Genome Project**
**http://zebrafish.stanford.edu/genome/Frontpage.html**
This page has genetic maps of zebrafish and the results of a comparison made with the human genome. There are also pages detailing the group's efforts towards the generation of new markers for use in the maps.

**The Zebrafish Deletion Project**
**http://www.ciwemb.edu/labs/halpern/zdp.html**
Although these pages are still under construction, there is a tool available to look for deletions available in each linkage group and contact details for members of the group, to arrange to obtain mutants from their stocks.

**The UCL Zebrafish Research Group**
**http://www.ucl.ac.uk/zebrafish-group/**
The group is involved in screening mutants from both of the Tübingen zebrafish mutagenesis projects. They are also undertaking a smaller-scale mutagenesis screen of their own. There is also a small stock centre, with just over 100 lines, which are made available to the community.

Copyright © 2000 John Wiley & Sons, Ltd.

### Zebrafish books

### General

Detrich HW, Zon LI, Westerfield M (eds). 1998. *The Zebrafish, vol 1: Biology*. Methods in Cell Biology Series. Academic Press: New York.

Detrich HW, Zon LI, Westerfield M (eds). 1998. *The Zebrafish, vol 2: Genetics and Genomics*. Methods in Cell Biology Series. Academic Press: New York.

### Techniques

Guille M (ed.). 1999. *Molecular Methods in Developmental Biology: Xenopus and Zebrafish*. Methods in Molecular Biology, vol 127. Humana Press: Totowa, NJ.

Westerfield M. 1994. *The Zebrafish Book: A Guide for the Laboratory Use of Zebrafish (Danio rerio)*. Institute of Neuroscience: University of Oregon.

### References

Amatruda JF, Zon LI. 1999. Dissecting hematopoiesis and disease using the zebrafish. *Dev Biol* **216**: 1–15.

Amemiya CT, Zon LI. 1999. Generation of a zebrafish P1 artificial chromosome library. *Genomics* **58**: 211–213.

Amores A, Force A, Yan Y-L, *et al.* 1998. Zebrafish *hox* clusters and vertebrate genome evolution. *Science* **282**: 1711–1714.

Aparicio S. 2000. Vertebrate evolution – recent perspectives from fish. *Trends Genet* **16**: 54–56.

Ardouin O, Legouis R, Fasano L, *et al.* 2000. Characterisation of the two zebrafish orthologues of the KAL-1 gene underlying X chromosome-linked Kallmann syndrome. *Mechanisms Dev* **90**: 89–94.

Driever W, Solnica-Krezel L, Schier AF, *et al.* 1996. A genetic screen for mutations affecting embryogenesis in zebrafish. *Development* **123**: 37–46.

Drummond IA. 2000. The zebrafish pronephros: a genetic system for studies of kidney development. *Pediat Nephrol* **14**: 428–435.

Fritz A, Rozowski M, Walker C, Westerfield M. 1996. Identification of selected $\gamma$-ray induced deficiencies in zebrafish using multiplex polymerase chain reaction. *Genetics* **144**: 1735–1745.

Geisler R, Rauch GJ, Baier H, *et al.* 1999. A radiation hybrid map of the zebrafish genome. *Nature Genet* **23**: 86–89.

Haffter P, Granato M, Brand M, *et al.* 1996. The identification of genes with unique and essential functions in the development of the zebrafish, *Danio rerio*. *Development* **123**: 1–36.

Hukriede NA, Joly L, Tsang M, *et al.* 1999. Radiation hybrid mapping of the zebrafish genome. *Proc Natl Acad Sci U S A* **96**: 9745–9750.

Hyatt TM, Ekker SC. 1999. Vectors and techniques for ectopic gene expression in zebrafish. *Methods Cell Biol* **59**: 117–128.

Johnson SL, Gates MA, Johnson M, *et al.* 1996. Centromere-linkage analysis and consolidation of the zebrafish genetic map. *Genetics* **142**: 1277–1288.

Kelly PD, Chu F, Woods IG, *et al.* 2000. Genetic linkage mapping of zebrafish genes and ESTs. *Genome Res* **10**: 558–567.

Krauss S, Concordet JP, Ingham PW. 1993. A functionally conserved homology of the *Drosophila* segment polarity gene-hh is expressed in tissues with polarizing activity in zebrafish embryos. *Cell* **75**: 1431–1444.

Kwok C, Korn RM, Davis ME, *et al.* 1998. Characterization of whole genome radiation hybrid mapping resources for non-mammalian vertebrates. *Nucleic Acids Res* **26**: 3562–3566.

Lacroix L, Arimondo PB, Takasugi M, Helene C, Mergny JL. 2000. Pyrimidine morpholino oligonucleotides form a stable triple helix in the absence of magnesium ions. *Biochem Biophys Res Comm* **270**: 363–369.

Lekven AC, Helde KA, Thorpe CJ, Rooke R, Moon RT. 2000. Reverse genetics in zebrafish. *Physiol Genom* **2**: 37–48.

Li YX, Farrell MJ, Liu RP, Mohanty N, Kirby ML. 2000. Double-stranded RNA injection produces null phenotypes in zebrafish. *Dev Biol* **217**: 394–405.

Linney E, Hardison NL, Lonze BE, Lyons S, DiNapoli L. 1999. Transgene expression in zebrafish: a comparison of retroviral-, vector and DNA-injection approaches. *Dev Biol* **213**: 207–216.

Malicki J, Neuhauss SCF, Schier AF, *et al.* 1996. Mutations affecting development of the zebrafish retina. *Development* **123**: 263–273.

Postlethwait JH, Yan Y-L, Gates M, *et al.* 1998. Vertebrate genome evolution and the zebrafish gene map. *Nature Genet* **18**: 345–349.

Saszik S, Bilotta J, Givin CM. 2000. ERG assessment of zebrafish retinal development. *Vis Neurosci* **16**: 881–888.

Scheer N, CamposOrtega JA. 1999. Use of the Gal4–UAS technique for targeted gene expression in the zebrafish. *Mech Dev* **80**: 153–158.

Shimoda N, Knapik EW, Ziniti J, *et al.* 1999. Zebrafish genetic map with 2000 microsatellite markers. *Genomics* **58**: 219–232.

Stainier D, Fouquet B, Chen J, *et al.* 1996. Mutations affecting the formation and function of the cardiovascular system in the zebrafish embryo. *Development* **123**: 285–292.

Wargelius A, Ellingsen S, Fjose A. 1999. Double-stranded RNA induces specific developmental defects in zebrafish embryos. *Biochem Biophys Res Comm* **263**: 156–161.

Whitfield TT, Granato M, van Eeden FJM, *et al.* 1996. Mutations affecting development of the zebrafish inner ear and lateral line. *Development* **123**: 241–254.

Yan YL, Talbot WS, Egan ES, Postlethwait JH. 1998. Mutant rescue by BAC clone injection in zebrafish. *Genomics* **50**: 287–289.

Zhong TP, Kaphingst K, Akella U, Haldi M, Lander ES, Fishman MC. 1998. Zebrafish genomic library in yeast artificial chromosomes. *Genomics* **48**: 136–138.

Copyright © 2000 John Wiley & Sons, Ltd.

*Comparative and Functional Genomics* is a cross-organism journal, publishing studies on complex and model organisms. The 'Featured Organism' article aims to present an overview of an organism, primarily for those working on other systems. It provides background information on the organism itself and on genomics studies currently in progress. It also gives a list of web sites containing further information and a summary of the status of the study of the genome. These sections are a personal critical analysis of the current studies of the particular organism. The 'Future Aims' section is intended to be of interest to readers who work on the chosen organism and those who study other systems, and the opinions expressed therein are those of the named contributors.

Many thanks to Professor Philip Ingham (Section Editor – zebrafish) for his critical appraisal of this article and to Professor Mark Fishman and Dr Stephen Wilson for sharing their thoughts on the future of zebrafish genomics research with me.

**Professor Mark Fishman**
Cardiovascular Research Center, MGH-East 4th Floor, 149 Thirteenth Street, Charlestown, MA 02129, USA. Tel: 0101 617 726 3738.
E-mail: fishman@cvrc.mgh.harvard.edu
**http://cvrc.mgh.harvard.edu/research/fishman/index.html**

**Dr Stephen Wilson**
Department of Anatomy and Developmental Biology, University College London, Gower Street, London WC1E 6BT, UK.
E-mail: s.wilson@ucl.ac.uk
**www.ucl.ac.uk/zebrafish-group/**

Copyright © 2000 John Wiley & Sons, Ltd.
